# Three-Way Analysis of Spectrospatial Electromyography Data: Classification and Interpretation

**DOI:** 10.1371/journal.pone.0127231

**Published:** 2015-06-03

**Authors:** Jukka-Pekka Kauppi, Janne Hahne, Klaus-Robert Müller, Aapo Hyvärinen

**Affiliations:** 1 Dept. of Computer Science/HIIT, University of Helsinki, Helsinki, Finland; 2 Brain Research Unit, O.V. Lounasmaa Laboratory, Aalto University, Espoo, Finland; 3 Dept. of Computer Science, Machine Learning Group, Berlin Institute of Technology, Berlin, Germany; 4 Dept. of Neurorehabilitation Engineering, Universitätsmedizin Göttingen, Göttingen, Germany; 5 Dept. of Brain and Cognitive Engineering, Korea University, Seoul, Korea; 6 Dept. of Dynamic Brain Imaging, Advanced Telecommunication Research Institute International (ATR), Kyoto, Japan; University of Texas School of Public Health, UNITED STATES

## Abstract

Classifying multivariate electromyography (EMG) data is an important problem in prosthesis control as well as in neurophysiological studies and diagnosis. With modern high-density EMG sensor technology, it is possible to capture the rich spectrospatial structure of the myoelectric activity. We hypothesize that multi-way machine learning methods can efficiently utilize this structure in classification as well as reveal interesting patterns in it. To this end, we investigate the suitability of existing three-way classification methods to EMG-based hand movement classification in spectrospatial domain, as well as extend these methods by sparsification and regularization. We propose to use Fourier-domain independent component analysis as preprocessing to improve classification and interpretability of the results. In high-density EMG experiments on hand movements across 10 subjects, three-way classification yielded higher average performance compared with state-of-the art classification based on temporal features, suggesting that the three-way analysis approach can efficiently utilize detailed spectrospatial information of high-density EMG. Phase and amplitude patterns of features selected by the classifier in finger-movement data were found to be consistent with known physiology. Thus, our approach can accurately resolve hand and finger movements on the basis of detailed spectrospatial information, and at the same time allows for physiological interpretation of the results.

## Introduction

Experimental biomedical data have an intrinsic structure in which data “points” are often matrix valued, i.e. the data should be essentially seen as a multi-way structure. After data collection, temporal measurements are cut to shorter time-windows, also called epochs, and then arranged into a spatio-temporal data array (time × space × epochs). Such three-(rsp. multi-)way arrays are common for instance in electromyography (EMG) [1, 2], magnetoencephalography (MEG) [3, 4], electroencephalography (EEG) [5–7] or functional magnetic resonance imaging (fMRI) [8]. Also multimodal data [9] may be arranged in such an array structure. Note that a representation that would squash multi-way data into vectorial shape would clearly ignore the intrinsic spatio-temporal dependency structure induced by the experimental setting and thus miss important information [8, 10, 11].

Applying machine learning methods on multi-way data is often not straight-forward due to these various dependencies, and therefore it is important to study the performance and properties of algorithms in this setting. One task in the analysis of multi-way data sets is to learn an association of the epochs to multiple categories (labels). As alluded above, a three-way classification problem differs from conventional multivariate classification, because each epoch is a matrix (tensor) instead of a vector. Utilizing standard classification methods, such as linear discriminant analysis (LDA) or linear/nonlinear support vector machines (SVMs; see for instance [5]) on vectorized data can yield suboptimal performance due to 1) the very high dimension of the data vector, and 2) the strong dependencies involved in both the signal and noise. Classifying data with special three-way methods which directly use the matrix structure can help to improve performance [10, 12, 13]. For example, bilinear models have been used successfully in the context of brain-computer interfaces [13]. A bilinear classifier is a model that assumes a simplified separable dependency structure typically in space and time.

It is common to enhance the analysis of biomedical data by applying different multivariate techniques, such as principal component analysis (PCA) [14, 15], common spatial pattern (CSP) filtering [1, 16], independent component analysis (ICA) [4, 17–21] and related blind source separation methods [22], to extract signal components with specific spatial characteristics. It is important to note that after such preprocessing, the use of dedicated three-way data analysis methods can still be highly useful, as will be clear below.

In this paper, we concentrate on the classification of three-way data from experimental studies using high-density EMG. High-density electrode arrays are particularly useful for decoding complex hand and/or finger movements, because these arrays can capture information from a high number of signal sources that get activated during corresponding contractions. In contrast to traditional electrode configurations based on only few EMG electrodes, a high-density EMG also allows for estimation of a generative model of source signal activity with much better spatial accuracy [17, 23].

EMG signals are produced by muscle fibers during contraction, and can be used to extract control signals from humans and to give insight to the neuromuscular system [24]. The generation of EMG signals starts by motor commands which are transmitted from the central nervous system to the muscles via action potentials on motoneurons. At the neuromuscular junctions, which are located within the innervation zone typically in the middle of muscle, the action potentials cause depolarizations of muscle cells belonging to one motor unit. These motor unit action potentials (MUAPs) propagate with a speed of approximately 4 m/s towards both ends of the muscle and cause a contraction on the fibers. Due to this propagation, slightly delayed versions of the MUAPs are detected at different channels within the electrode-arrays used in this study. Therefore, a generative model for describing the detected signals should preferably be convolutive [17]. Convolutive models have been previously developed for EEG and MEG data to model phase-lagged activity in different brain areas [19, 25]. In particular, the method called “Fourier-ICA” [19] was developed to model spatially extended sources of rhythmic brain activity in the time-frequency domain with constant phase shifts. Thus, we assume that the Fourier-ICA is capable of detecting the propagation of the MUAPs in high-density EMG arrays due to relatively slow propagation speed of the MUAPs and high spatial resolution of such arrays.

After an amputation, the patient often retains a so called phantom feeling for the missing limb, which he/she can still actuate. For instance, in case of transradial amputation, usually large parts of the forearm muscles that used to control the fingers and wrist remain intact and contract when the phantom limb is actuated. Since the generated EMG signals reflect the muscle-forces, they can be used to extract control information, e.g. to actuate a forearm prosthesis [26]. Most research on this topic has so far focused on relatively simple signal processing schemata where features are extracted from the segmented signals of typically less than ten bipolar electrodes and a subsequent classifier learns the type of movement. Such a procedure is mainly used to extract rough movements such as hand gestures and wrist movements. Modern high-density EMG gives high-dimensional data which has potentially much more information, but extracting such new information may necessitate more sophisticated methods.

Our goal in this contribution is to investigate the suitability of three-way methods for EMG signal classification. We improve and adapt existing methods to make them better fit high-density EMG and its rich spectro-spatial structure. We aim at building a high-accuracy classifier that, importantly, also provides meaningful insight into high-dimensional, three-way EMG data and thus enables a simple interpretation of the physiology behind the classification process. In particular, we first apply the Fourier-ICA algorithm to estimate independent components from the multiarray EMG measurements. Then, we combine a sparsity penalty with a bilinear classifier to improve classification and interpretability of the method. We furthermore develop a computationally efficient and statistically stable extension of linear discriminant analysis to three-way data. In sum, these advances contribute to a significant progress over the existing state-of-the-art in EMG analysis.

## Materials and Methods

### EMG data

We analyzed data sets from two different EMG movement experiments. The experiments were in accordance with the declaration of Helsinki and were approved by the local ethics commission (Ethikkommission der Charité Berlin, approval number: EA4/085/11). A written informed consent, which was approved by the ethics commission, was obtained from each participant.

#### Hand-movement data

The first data set was collected previously [1] in an experiment where ten able-bodied subjects repeatedly performed six different hand contractions (hand open, hand close, pronation, supination, wrist flexion, and wrist extension) in five different arm positions. Each contraction lasted about 3 s and was performed with approximately 40% force. Monopolar EMG signals from 96 channels were recorded from the forearms of the subjects using a multiarray EMG grid. For more details of the data and experiment, please see [1]. Our goal was to classify the data set into the six aforementioned movement categories (different arm positions were combined together). We used this data set to quantitatively evaluate the performance of the classifiers.

#### Finger-movement data

The second data set was collected particularly for this study to demonstrate that our methods are capable of exploiting propagating signal-components that are caused by the underlying composition of the EMG from individual MUAPs. We chose the investigation of individual finger-movements that involve relatively small and adjacent but also superficial muscles located in the forearm. This is not only a challenging classification task which is highly relevant for advanced hand-prostheses, but also an interesting example to demonstrate the interpretability of the generated patterns. We wanted to collect new and interesting data for the purpose of interpretation, because the hand-movement data set has already been examined previously [1].

**Fig 1:**
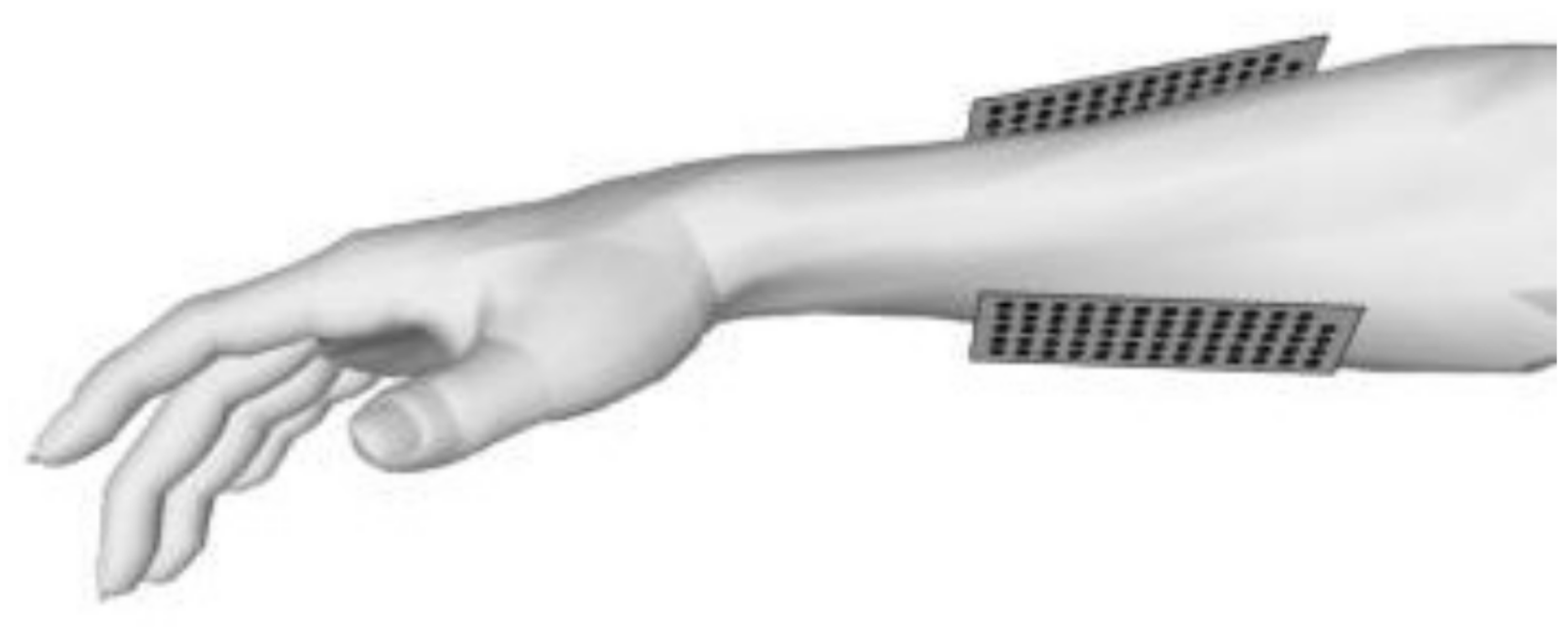
The placement of the high-density EMG sensors in the finger movement experiment.

We recorded 128 monopolar EMG signals from the right hand of one subject, using four “Brain Amp DC” biosignal amplifiers at a sampling frequency of 2500 Hz. This sampling frequency was used to avoid aliasing of the signal (frequency range approx. 0–500 Hz) and to allow for digital filtering. Two Ag/AgCl matrix electrode arrays with an inter-electrode distance of 8 mm (BioOT-ELSCH064R3S) were placed on the skin above the extensors and the flexors of the right (dominant) forearm. The arrays were attached to the skin with foam-pads that contain holes, filled with conductive electrode-paste. They were placed on the forearm (approximately 1/3 from elbow towards the wrist), directly above the muscles that are responsible for the contractions investigated in this study (see Fig 1).

The subject was instructed to perform both isometric flexions (pressings) and extensions (liftings) of the five individual fingers, which resulted in 10 different classes or categories. Further, the subject was instructed to alternate between three different force levels (low/medium/high). Each trial lasted for three seconds. Accurate data labeling was ensured by software that presented the instructions and some feedback to the subject on a computer screen. The subject performed a total of 300 contractions (10 classes, 3 force levels, 10 repetitions). All force levels were combined together in classifier training and testing to mimic variabilities that are present in typical EMG applications.

#### Preprocessing

Before classification, the data was temporally filtered with a 4th order Butterworth bandpass filter (*f*_*low*_ = 20 Hz, *f*_*high*_ = 500 Hz) and a 50 Hz comb-filter to remove movement-artifacts, noise above the EMG band and power-line interferences. The amplitude of the filtered EMG for high contractions was in the range of 185 *μV*_*RMS*_. We performed classification based on 200-ms non-overlapping epochs. For this purpose, we organized hand and finger movement data sets into a three-way spatio-temporal array (time × channels × epochs). This lead to 480 epochs per category for the finger movement data and, depending on the subject, 120–210 epochs per category for the hand movement data.

### Overview of low-rank bilinear logistic regression

Let us denote a set of epochs (time windows) recorded from multiple sensors in matrix form as:

$$\begin{array}{c} X\left(n\right)={\left[x_{1}\left(n\right),\;x_{2}\left(n\right),\;\ldots \;x_{J}\left(n\right)\right]}^{T}\in {\mathbb{R}}^{D\times E},\text{for}\;n=1,\;2,\;\ldots ,\;N, \end{array}$$ (1)

where the matrix **X**(*n*) denotes the *n*th epoch, the vector **x**_*i*_(*n*) contains the measurements (time points) from the *i*-th sensor for the *n*-th epoch, *E* is the total number of sensors, *D* is the number of time-points in one epoch and *N* is the total number of epochs in the data set. In practice, each epoch may also have been preprocessed by Fourier transformation, replacing the time index by the frequency index.

A simple approach to estimate parameters of the matrix classifier would be to transform matrices to vectors before classification, defining the regression (classifier) function *K* by:

$$\begin{array}{c} K\left(G;X\right)=\left\langle G,X\right\rangle =\text{vec}\;{\left(G\right)}^{T}\;\text{vec}\;(X), \end{array}$$ (2)

where **G** ∈ ℝ^*D*×*E*^ denotes the parameter matrix, ⟨⋅, ⋅⟩ is the elementwise inner product between two matrices, and vec(⋅) denotes the vectorization of the matrix columnwise. Because the above function is linear in its parameters, a linear classifier can be trained on these vectors. However, this approach does not utilize the specific structure of the feature matrices and may lead to infeasible parameter estimation problem due to high number of model parameters. To improve the classifier, one can impose a rank constraint on the parameter matrix, such that **G** = **W** **V** ^*T*^, where **W** = [**w**_1_ **w**_2_ … **w**_*R*_] ∈ ℝ^*D*×*R*^ and **V** = [**v**_1_ **v**_2_ … **v**_*R*_] ∈ ℝ^*E*×*R*^ are two separable parameter matrices of rank *R* where *R* is small. In this case, we have the following *bilinear regression function*:

$$\begin{array}{c} K(W,V;X)=\left\langle WV^{T},X\right\rangle =\text{Tr}\;({\left({\text{WV}}^{T}\right)}^{T}X)=\text{Tr}\;(W^{T}XV). \end{array}$$ (3)

A binomial logistic regression (LR) can be used to optimize **W**, **V** for binary classification [13]. The binomial LR model is given by:

$$\begin{array}{c} P\left(y=1|X\right)=\frac{1}{1+\exp(K(W,V;X)+b)} \end{array}$$ (4)

where *P*(*y* = 1|**X**) is a posterior probability that data matrix **X** belongs to category 1, and *b* is a bias term. Under the assumption that target variables (labels) *y*_*i*_ ∈ {0, 1} are independent and follow a binomial distribution, the normalized log-likelihood of the model is given by:

$$\begin{array}{ccc} \theta_{W,V} & = & \frac{1}{N}\sum_{i=1}^{N}\;(y_{i}\left(K(W,V;X(i))+b\right)-\log(1+e^{K(W,V;X(i))+b})). \end{array}$$ (5)

### Sparse low-rank bilinear logistic regression

While the bilinear classifier provides a useful approach to three-way data classification, it does not use the full power of modern learning theory. In particular, sparsity has been shown to improve classification in many forms and many domains. For instance, benefits of utilizing sparsity in continuous arm prosthesis control on the basis of multiarray EMG signals were recently demonstrated [27].

In fact, there is also a deeper reason why a sparsity penalty or similar should be extremely useful for a proper interpretation of bilinear classification. As already pointed out by [13], the solution is highly non-unique, since we have

$$\begin{array}{c} \text{Tr}\;(W^{T}XV)=\text{Tr}\;(UW^{T}XVU^{-1}) \end{array}$$ (6)

for any invertible matrix **U**. Thus, the interpretation of the obtained classifier matrices **W** and **V** is not possible without specifying a principled way of finding the transformation **U**. We propose to use the sparsity of the classifier matrices of interest for this purpose.

Thus, we introduce a sparsity penalty on **W** and/or **V** in the likelihood, and maximize the following objective:

$$\begin{array}{c} \max_{W,V}\;\{\theta_{W,V}+\lambda^{w}Lp_{W}\left(W\right)+\lambda^{v}Lp_{V}\left(V\right)\}, \end{array}$$ (7)

where *Lp*_**W**_(**W**) and *Lp*_**V**_(**V**) are regularization terms and at least one of them is the sparsity penalty of the form: *L*_1_(**Z**) = ∑_*ij*_|*z*_*ij*_|. Constants *λ* ^*w*^ and *λ* ^*v*^ control the amount of regularization and their values are selected through cross-validation. We call the model sparse (low-rank) bilinear classifier.

Optimization of this objective can be difficult due to the *L*_1_-norm penalty functions which are non-differentiable. However, there exist computationally efficient algorithms for sparse estimation of the linear regression models, suggesting that we can use an “alternating” strategy to maximize the above penalized likelihood. In this strategy, we optimize one parameter matrix at a time while keeping another parameter matrix fixed. For instance, by keeping **V** fixed, we can rewrite the regression function as a linear function of **W**:

$$\begin{array}{c} K\;(W;V,X)=\text{Tr}\;(W^{T}XV)=\text{Tr}\;(W^{T}Y)=\left\langle W,Y\right\rangle \end{array}$$ (8)

where **Y** = **X** **V**. Similarly, when **W** is fixed, we obtain a linear function of **V**. Taking into account the two sparse penalization terms, we actually have the same objectives as in logistic LASSO [28]. If one of the terms is sparse and another one is the traditional *L*_2_-norm, ridge regression and LASSO problems need to be solved alternately.

To estimate the sparse low-rank bilinear classifier, we can use existing optimization algorithms [29–31] to obtain estimates of the parameter matrices for a sequence of regularization parameters. To find the best regularization parameters, we need to estimate the model for all the possible *m* × *n* regularization parameter pairs $\left(\lambda_{i}^{v},\lambda_{j}^{w}\right)$ where *i* = 1, …, *m* and *j* = 1, …, *n*. Putting all these together, our method is as follows:

1. Set rank constraint *R* and initialize ${\text{V}}_{\lambda_{i}^{v}}$, for $\lambda_{1}^{v},\lambda_{2}^{v},\ldots ,\lambda_{m}^{v}$ to random values.
2. For each ${\text{V}}_{\lambda_{i}^{v}}$ fixed, estimate ${\text{W}}_{\lambda_{j}^{w}}$, for $\lambda_{1}^{w},\lambda_{2}^{w},\ldots ,\lambda_{n}^{w}$ by maximizing (5) with respect to **W** only, using the existing methods.
3. For each ${\text{W}}_{\lambda_{j}^{w}}$ fixed, estimate ${\text{V}}_{\lambda_{i}^{v}}$ for $\lambda_{1}^{v},\lambda_{2}^{v},\ldots ,\lambda_{m}^{v}$ likewise.
4. Alternate the above two steps until parameter estimates converge. Then, evaluate the bilinear model for all pairs $\left({\text{V}}_{\lambda_{i}^{v}},{\text{W}}_{\lambda_{j}^{w}}\right)$ using a separate validation data set and pick the best parameters.
5. Compute the test accuracy for an independent data set using the selected model (regularization parameters and classifier parameters) and visualize the classifier.

Thus we obtain a simple method for incorporating a sparsity prior in bilinear classification, utilizing the full power of recently developed sparse classifiers. Generalization for multi-category problems is straightforward using well-known principles, such as one-versus-the-rest (OVR) classification.

#### Fourier-domain independent component analysis

One of our motivations for using a sparsity enforcing classifier is that when combined with a suitable decomposition (representation) learning method, the classifier allows for the automatic selection of the relevant signal components for physiological investigation. Next, we motivate the ICA decomposition of original raw EMG measurements and propose a method for learning such a decomposition. ICA is a multivariate technique that has been widely used to decompose different physiological signals into “source” components [32]. The basic assumptions of the ICA model are that 1) the measured signals are a linear mixture of the source components, 2) the sources are independent and non-Gaussian, and 3) the mixing is instantaneous, meaning that a source signal is observed simultaneously in each sensor. Since the components given by ICA are maximally independent, it is likely that the method isolates the information crucial for the classification to a small number of components. Thus, the sparsity-enforcing classifiers are likely to work better when applied on the independent components (ICs) than when applied on the original channels [4].

It has been shown previously that ICA applied in temporal domain of the EMG signal can exploit a rich correlation structure of the multiarray EMG and provide valuable information from these data [17]. However, a major limitation of the basic ICA model in the context of EMG signal analysis is the assumption of instantaneous mixing. In practice, as the MUAPs in muscles are propagating along the muscle-fibers, they are detected in slightly different time on different sensors within the electrode array (in longitudinal direction with respect to the fibers). Therefore it would be better to use a convolutive ICA model, which takes these time-delays into account, to analyze EMG. Previously, the method called Fourier-ICA [19] was developed to model spatially extended sources of rhythmic brain activity in the EEG/MEG signals with constant phase shifts. Because of the high spatial resolution of the high-density EMG and relatively slow propagation speed of the MUAPs, we assume that Fourier-ICA is capable of revealing the propagation of the MUAPs. Fourier-ICA is carried out in the time-frequency domain of the signal and thus allows separate analysis of the magnitudes and phases of the independent components, and in particular it allows for phase shifts (time delays) between the different sensors.

Next, we explain the details of the ICA estimation procedure. At first, we Fourier-transformed the spatio-temporal matrices (time × channels × epochs) into spectro-spatial matrices (frequency × channels × epochs) in which each column contains the Fourier-coefficients of the sensor in that window. Then, we performed ICA in the Fourier-domain following [19]. For the purpose of ICA, the three-way data was collapsed into an ordinary matrix by concatenating all the epochs and frequencies; the justification for such a procedure is presented in more detail in [19]. Complex-valued ICA was performed on this collapsed matrix (channels × time ⋅ frequency) to estimate ICs. No dimension reduction by PCA was performed. Likewise, the total number of estimated ICs corresponded to the total number of channels of the EMG sensor array.

The resulting ICs were rearranged back into a three-way data but whose dimensions were now: frequency × ICs × epochs. Finally, we took the absolute values of all the entries in the three-dimensional data tensor. This is because the tensor contains complex-valued Fourier coefficients, and conventional machine learning algorithms need real-valued input. We also assumed that most of the task-discriminative information is present in the amplitude spectra of the independent components. The phase information was only used in the final interpretation of the results.

#### Automatic selection of discriminative independent components

We thus combined Fourier-ICA and sparse bilinear classification to extract control information for prosthesis control as well as to automatically find physiologically interesting components from multiarray EMG data. In our study, the term “sparse” reflects the number of zero elements in the classifier parameter matrices **V**, **W** and not the statistical, non-Gaussian properties or the spatial magnitude patterns of the ICs. The inputs of the classifier are matrices corresponding to epochs (frequency × ICs), and the parameters to be estimated are the parameter matrix **W** for frequency coefficients and the parameter matrix **V** for the coefficients of the ICs. After classifier training, the final parameter matrix of the ICs contains non-zero elements only for those ICs that are discriminative in classification. This way, the method automatically returns a discriminative subset of ICs among many redundant and noisy ones. In a binary classification scenario, a classifier recovers a single subset of task-discriminative ICs. In a multicategory scenario, a separate subset of ICs can be recovered for each category by using e.g. the OVR scheme. Note that the total number of ICs in the estimated classifiers may vary notably between categories and subjects because the total number of non-zero parameters is automatically determined by the algorithm.

Due to its bilinear structure, our classifier makes a simplistic assumption that the estimated ICs share similar spectral characteristics. However, this assumption may not be as restrictive as it seems in the first sight, because it holds only for a (possibly small) subset of discriminative ICs selected by the classifier and not for all the estimated ICs prior to classification. Moreover, by increasing the rank R, other subsets of ICs with different spectral characteristics can be added to the model to improve its flexibility if needed.

### Three-way discriminant analysis for dimension reduction

An alternative approach to three-way data classification is to reduce the dimension of the data by computing a small number of components which are particularly relevant for classification, and using them in an ordinary classifier. A number of methods have been proposed recently [12, 33, 34]. Here, we propose a particularly simple and computationally efficient method based on the theory of Fisher’s linear discriminant analysis (LDA).

Considering binary classification, denote the difference of the class means by

$$\begin{array}{c} \Delta E=E\{X|y=1\}-E\{X|y=0\}, \end{array}$$

where *y* denotes the class labels. We propose to maximize

$$\begin{array}{c} \max_{∥w∥=∥v∥=1}w^{T}\;\left(\Delta E\right)v \end{array}$$

which is the basic definition of ordinary LDA with a) the assumption that the class covariances are all equal, and scalar times identity (isotropic) and b) the assumption that the separating vector has matrix form, and rank one as **w** **v** ^*T*^. This can be seen as a regularized version of [12], where the covariance matrix is regularized before inversion by adding an identity matrix to it with an infinite weight. Such regularization has the benefit that we completely avoid the computational problems and possible instability related to the inversion of the covariance matrix which is usually done in LDA.

In ordinary LDA, solving this optimization problem we get simply the vector which is the class difference. However, in the three-way case, the solution is given by the dominant singular vectors of Δ**E**. Moreover, in the three-way case, it is actually meaningful to compute *many* pairs of singular vectors, while in the ordinary (two-way) case a single vector exhausts all information contained in the class difference. In fact, we can optimize the same objective function again, constraining the new **w** and **v** to be orthogonal to the previous ones. The solutions are again given by the singular vectors [35].

Thus, we get the following method for binary classification of three-way data:

1. Compute the singular value decomposition (SVD) of Δ**E**, the matrix of the differences of the class means.
2. Take the *R* left (resp. right) singular vectors corresponding to the largest singular values, as the **w**_1_, …, **w**_*R*_ (resp. **v**_1_, …, **v**_*R*_).
3. Compute the *r*-th feature for the *n*-th data point as ${\text{w}}_{r}^{T}\text{X}(n){\text{v}}_{r}$.

Our approach provides a simple and fundamental way of reducing three-way data into ordinary two-way data. Any ordinary classifier can be used to classify the data based on the features computed above. We could also incorporate sparsity in this estimation process by using a sparse SVD method [35].

### Classifier comparison

We compared the performance of our sparse bilinear (denoted by “Bilinear”) and three-way DA (“ThreeWay DA”) methods against four other classifiers: *1)* An ordinary LDA classifier based on Hudgins features and PCA dimension reduction, where the optimal dimension is determined using the CV [36] (denoted by “Hudgins”; this is the state-of-the-art method used in classification of myoelectric signals and serves here as our baseline method); *2)* a linear LASSO classifier, for which we vectorized the matrix data prior to classification (denoted by “Linear”); *3)* the 2D-LDA dimension reduction method [12], again combined with LASSO-regularized linear logistic regression (denoted by “2D-LDA”); *4)* a linear classifier with a trace-norm penalty [10], which imposes a soft low-rank constraint on the parameter matrix (denoted by “TraceNorm”).

As Fourier-ICA is an integral part of our classifier design, we used it to preprocess the original signals prior to classification. The only classifier for which we did not apply Fourier-ICA was “Hudgins”, because it relies on characterists of temporal EMG signals which would not be detectable from the spectra of the ICs.

We employed five-fold cross-validation to estimate the classification accuracy. For the sparse bilinear classifier, we used the L1General Matlab software with the PSSG optimization [37] for classifier training and performed multicategory classification using the OVR scheme. The features of “ThreeWay DA” were extracted using the OVR scheme and classified using the LASSO-regularized linear logistic regression based on the dual augmented lagrangian algorithm [30]. We fixed the rank *R* = 1 in the estimation of both the three-way DA features and the sparse bilinear model in the comparative study, but we also evaluated the effect of the rank to the classification performance.

## Results

### Classification accuracy for hand movement data

**Fig 2:**
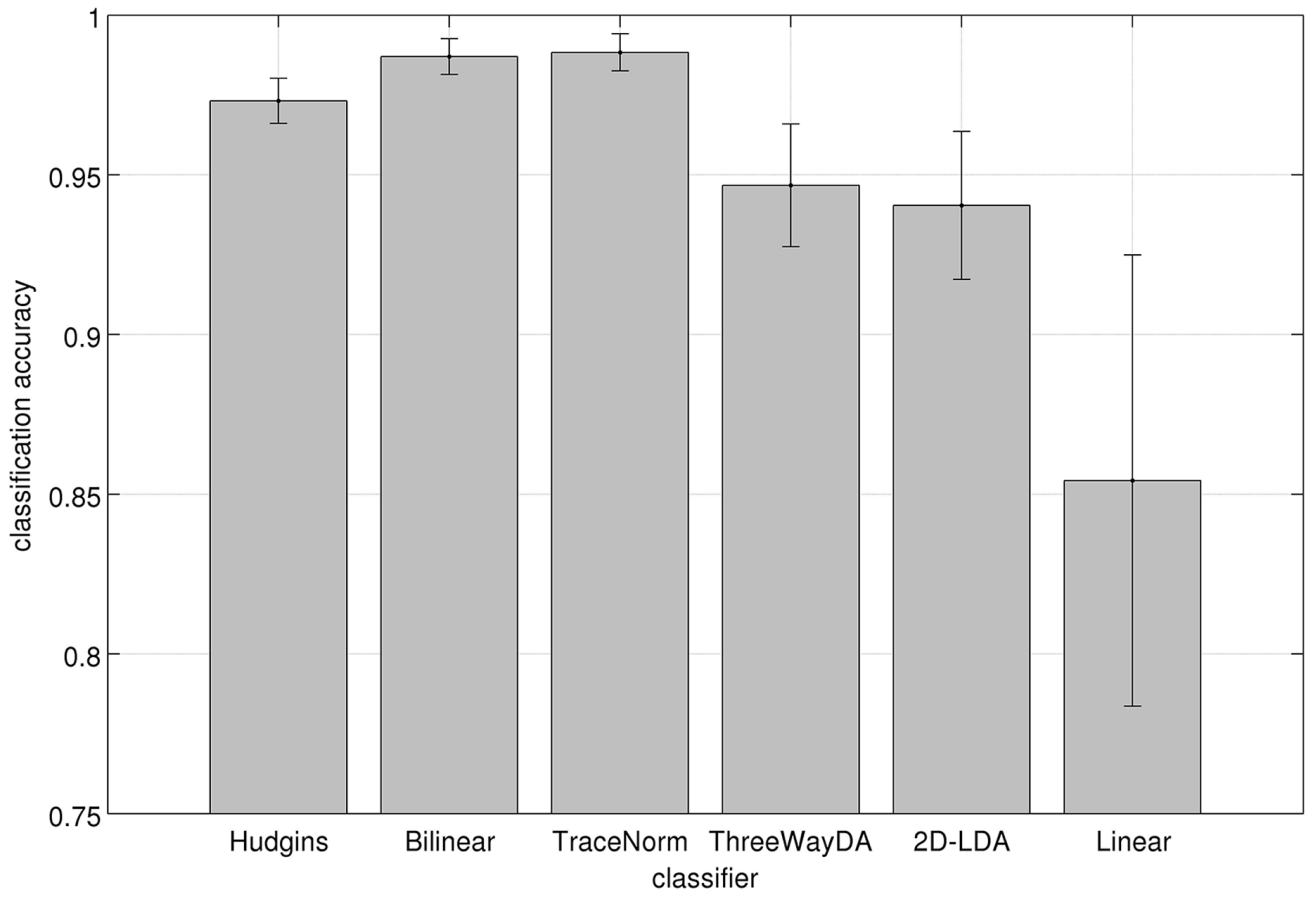
EMG classification results for the 6-category EMG hand movement task. Bars denote the mean (and the standard error of mean) of the classification accuracies across the subjects. The chance level of the classification was 0.167.

Fig 2 shows the results of the six tested classifiers for the 6-category hand movement data. Our “Bilinear” (the mean accuracy across subjects was 0.987) classifier yielded the best result together with the “TraceNorm” (the mean accuracy 0.988; according to the paired *t*-test, there was no statistically significant difference between the accuracies of these methods). The result of “Bilinear” was significantly better when compared with the baseline method “Hudgins” (paired *t*-test; *p* = 0.0019), which reached the mean accuracy of 0.973. The accuracies of the “ThreeWayDA” (mean accuracy 0.947) and “2D-LDA” (mean accuracy 0.940) were slightly worse than to that of the baseline method. The classifier “Linear” (mean accuracy 0.854) yielded the worst result among the tested methods.

**Fig 3:**
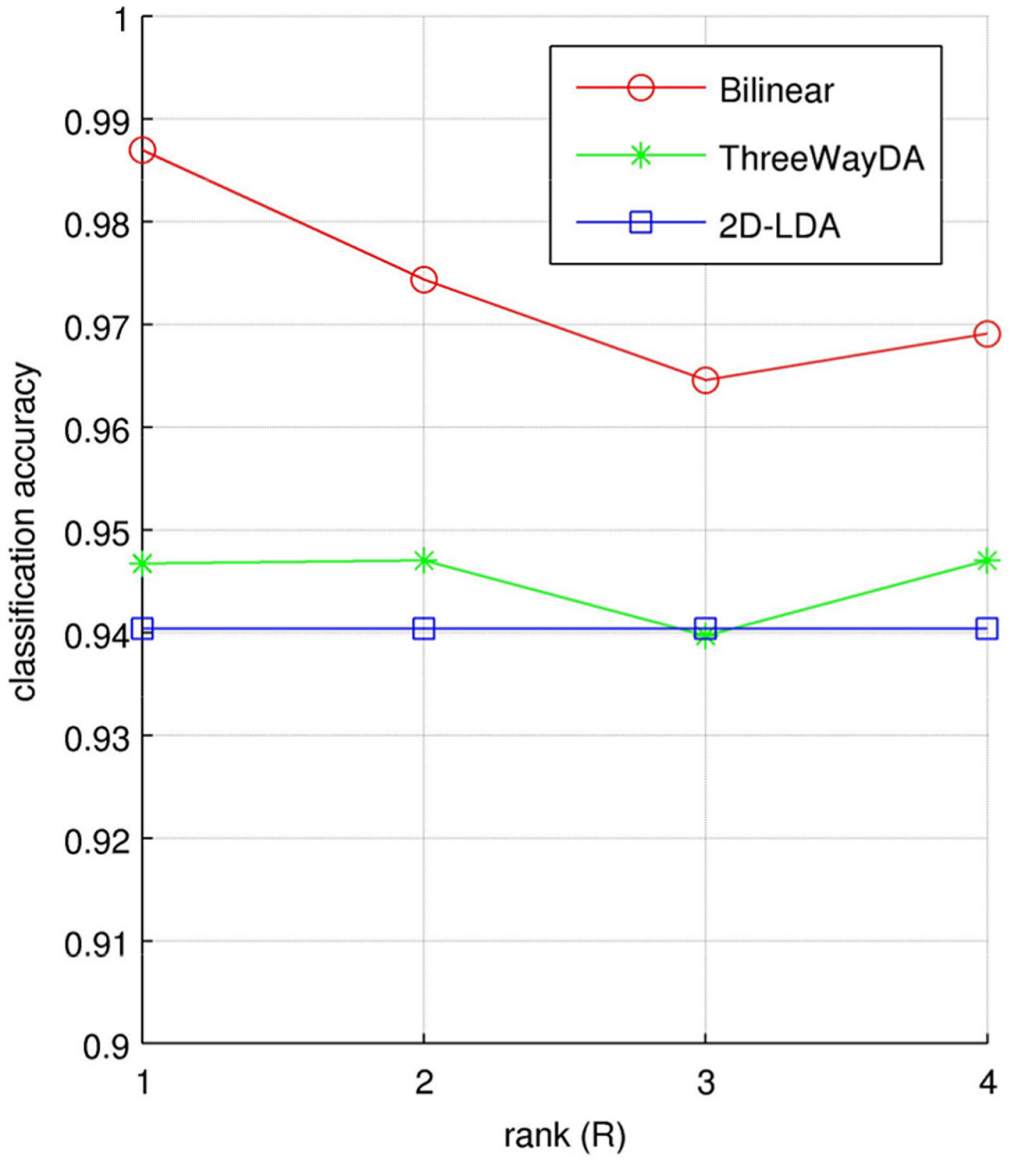
The effect of rank (R) to the classification accuracy for the hand movement data, using the methods “Bilinear”, “ThreeWayDA”, and “2D-LDA”.

We also evaluated how the choice of rank affects the performance of the classifiers. Fig 3 shows the effect of rank to the classification accuracy for the three relevant methods: “Bilinear”, “ThreeWayDA”, and “2D-LDA”. For the “Bilinear”, the best result was obtained with the rank = 1 and the results degraded slightly with the increase in the rank. For the “ThreeWayDA” and “2D-LDA”, the effect of rank to the classification accuracy was minimal.

### Interpretation of finger movement data

The high classification rates above show that three-way classification methods can efficiently utilize spectrospatial information of EMG signals. Next, we demonstrate how the proposed sparse bilinear classifier can be used to gain insights into high-dimensional finger movement EMG data (our second data set collected particularly for this purpose). The investigation of EMG activity during finger movements is important as it facilitates the understanding of neurological dysfunctions [38, 39] as well as the optimization of modern myoelectric prosthesis control systems allowing fine-grained movements [40, 41]. The main idea is to investigate the spatial patterns of ICs corresponding to non-zero IC coefficients (given in the columns of **V**) of the trained classifier. To ensure that the interpretation of finger movement data is meaningful, we validated that the bilinear classifier was successful also with this data set. For this purpose, we trained the classifier using 2/3 of the data, and evaluated the performance using the remaining 1/3 of the data. The median accuracy across all pairs of finger movement tasks was 0.98, showing that the parameter estimation was highly reliable.

**Fig 4:**
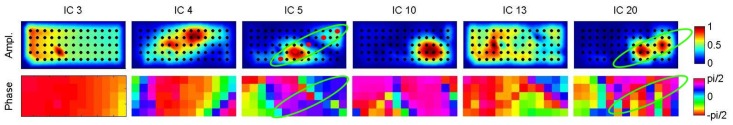
Magnitude and phase patterns of all discriminative ICs found with the sparse bilinear classifier in finger movement data, task “lift ring finger” versus “lift little finger”. The upper row shows the normalized amplitude pattern. Black dots mark the position of the sensors and values in between are obtained by bi-cubic interpolation. The lower row shows the corresponding phase information. Here no interpolation was done because of the periodicity of the phase. Note that phase information is only meaningful in locations where amplitude is non-zero. The sensors showing interesting phase distribution are denoted by the ellipses. The left side of the array is pointing towards the wrist, right side towards the elbow (see Fig 1).

**Fig 5:**
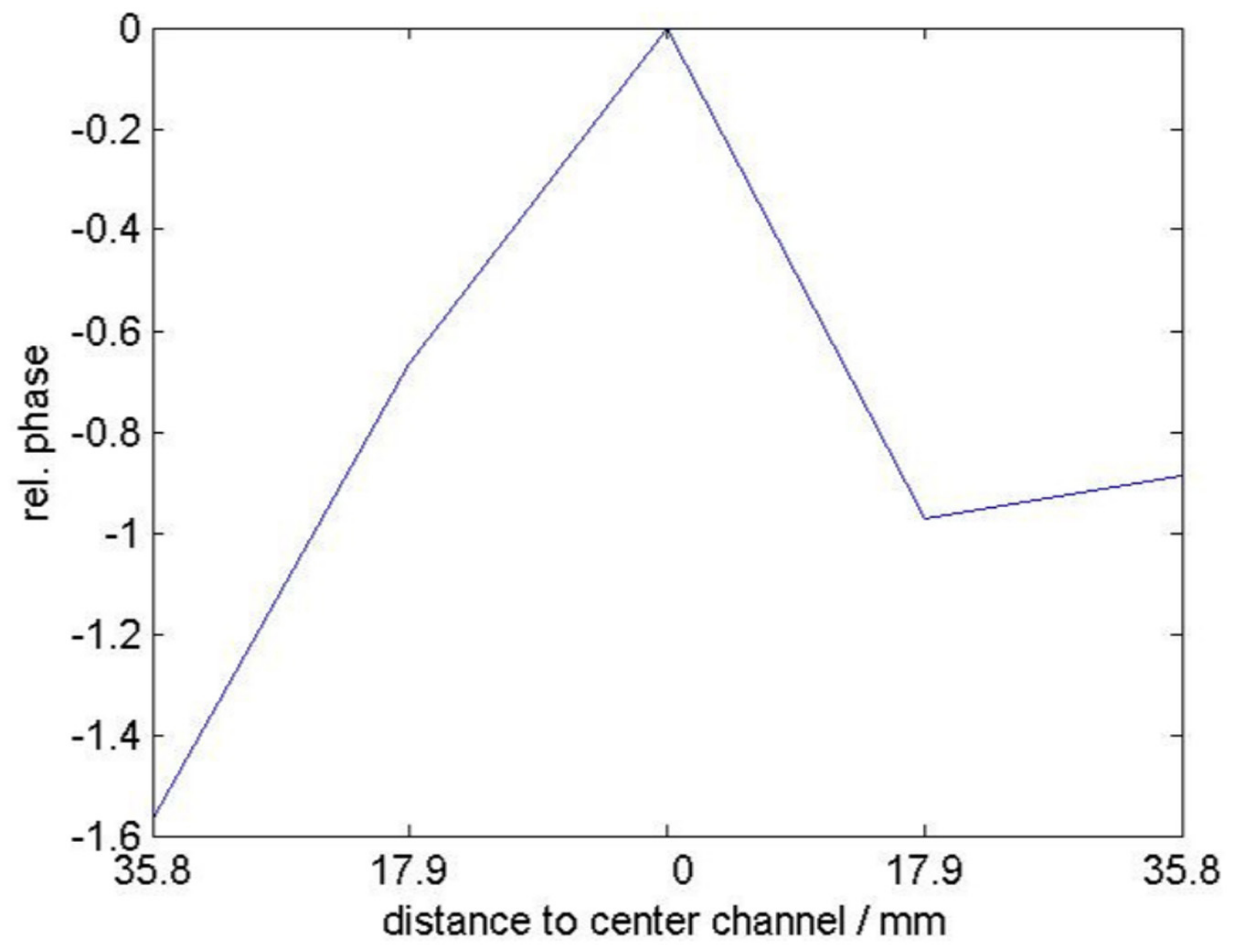
Phase information for five selected channels of IC #5 in Fig 4. The center channel is assumed to be located in the middle of the muscle close to the innervation zone. The x-axis shows the distance to the center channel and the y-axis shows the phase difference relative to the center channel. Assuming one dominant frequency the phase corresponds to a time delay and linear changing phase to propagation with constant speed.

From all the finger movement tasks, we selected one particular task, “lift ring finger” versus “lift little finger”, for closer inspection, by concentrating on the sensor array of 64 electrodes located above the extensors of the forearm. We expected that the analysis of this task is particularly interesting because the classifier must distinguish between activity in relatively small and adjacent muscles located near the sensor array. Fig 4 shows the spatial and phase pattern of the ICs correponding to non-zero classification coefficients of the sparse bilinear classifier for this task. When interpreting the findings, it is important to keep in mind that while the classifier returns a subset of components which maximally separate between the two categories, it does not mean that the components necessarily have meaningful neuroscientific interpretation, because also artefactual components may improve separability between the categories. For instance here, the amplitudes of ICs #3 and #13 have no clear structure and may be driven by noise. ICs #4,#5,#10 and #20 on the other hand clearly focus on spots that correspond to muscular activity under the sensors. For ICs #5 and #20 one can observe an interesting phase distribution in the sensors denoted by the ellipses: There is a relatively small phase in the center of the assumed muscle position and the phase changes gradually in both directions along the muscle. We hypothesize that this is because of the propagation of the MUAPs from the innervation zone towards the tendons. To investigate this in more detail for IC #5, the phase of some channels on a line parallel to the assumed direction of the muscle fibers (red channels) is shown in Fig 5. Starting from the center, the phase is changing proportionally with the distance to the center electrode with similar slope in both directions. Assuming one dominant frequency on which the ICs mainly react, this corresponds to propagation with constant velocity towards both sides. The fact that the phase does not further change for the last channel on the very right means that the end of the fiber is reached where the MUAPs remain without propagation for a short time before they vanish.

### Dimension reduction with three-way DA

**Fig 6:**
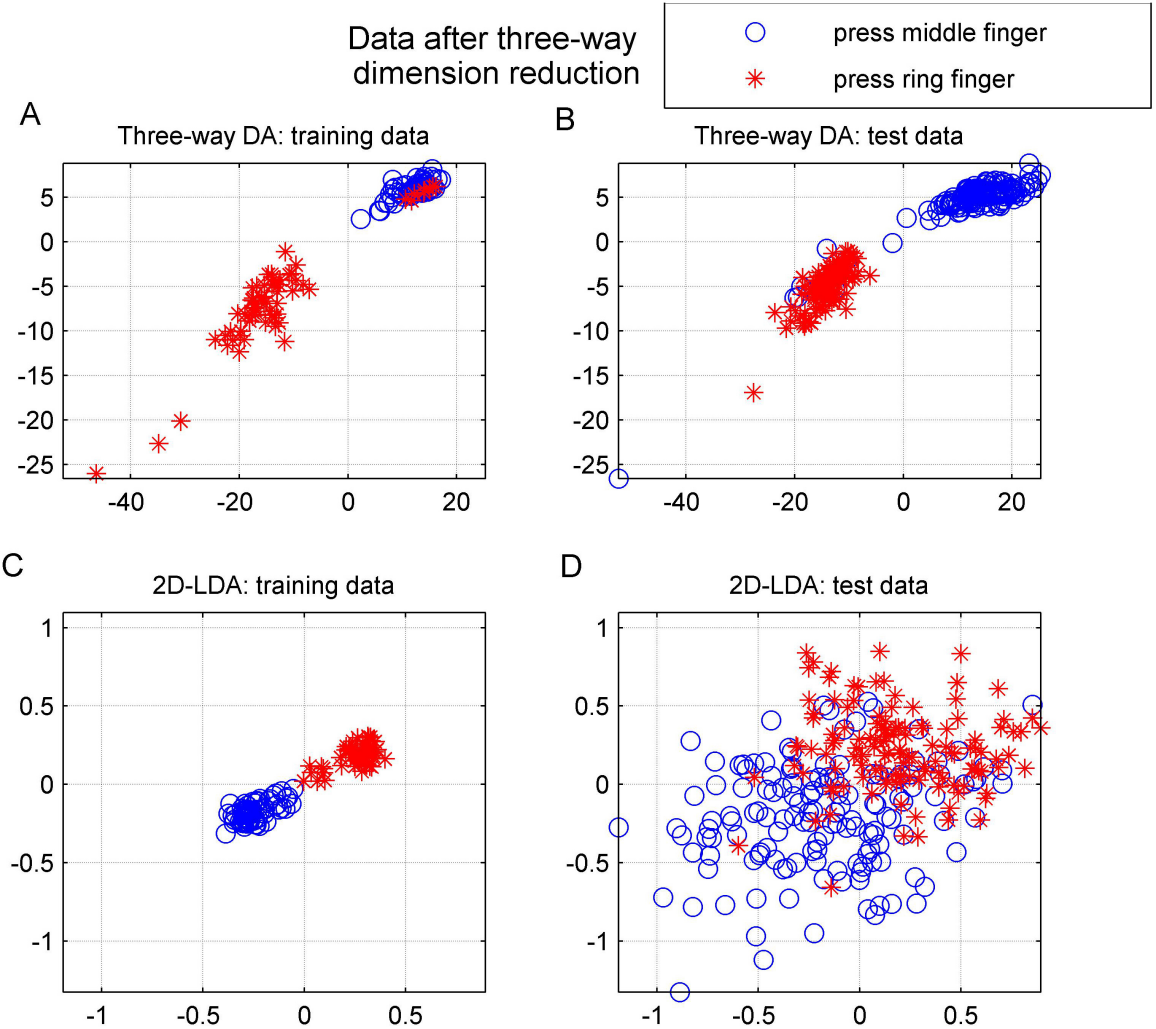
Dimension reduction from data matrix to 2-dimensional data: A) training data for “ThreeWayDA”, B) test data for “ThreeWayDA”, C) training data for “2D-LDA”, D) test data for “2D-LDA”. The horizontal axis corresponds to the first and the vertical axis to the second dimension.

Our second tensor method, “ThreeWayDA”, yielded reasonable classification accuracy. We next illustrate how this method can further provide a useful low-dimensional visualization of the EMG data. Figs 6A and 6B show the training and test sets after dimension reduction with the three-way DA for one arbitrarily selected binary task (“press middle finger” vs. “press ring finger”). We used the rank = 2 model to provide a 2-D representation of the original data. Clearly, the categories were well-separable in a two-dimensional feature space. In fact, the categories were well separable already in 1-D, i.e., the direction of the rank-1 approximation of the mean difference could separate the two classes in this case. The closer analysis of our classifier coefficients showed that the classifier selected a rank-1 solution (i.e., the second classification coefficient was enforced to zero by the LASSO). This example demonstrates that our method can be highly useful in simplification of high-dimensional tensorial EMG data. Because the proposed three-way DA can be seen as a regularized version of 2D-LDA dimension reduction method, it is interesting to compare the features of these methods visually. Figs 6C and 6D show the corresponding plots for 2D-LDA. This method overfitted the data: it provided excellent separation of the training data but the test data was not as well-separable as in the case of three-way DA.

## Discussion

Until now, ordinary two-way signal processing techniques have been used to analyze EMG; none of them employs the rich spectrospatial matrix structure that is present in the multi-way EMG. In this work, we proposed the use of three-way classification methods to multiarray EMG data, as well as contributed algorithmically to three-way classification. In particular, we adapted low-rank bilinear classification to EMG and combined it with sparseness penalties and Fourier-domain independent component analysis to analyze task-discriminative spectrospatial features of the EMG signal. Furthermore, we extended linear discriminant analysis to three-way data for transforming the three-way problem into an ordinary vector classification while at the same time preserving information contained in the multi-way structure. Besides classification, this method allows for visualization of complex high-density EMG data in a low-dimensional feature space.

Three-way classification applied on spectrospatial domain of the EMG signal turned out be highly successful. Especially, “TraceNorm” and “bilinear” yielded nearly 99% classification accuracy. This result suggests that multiarray EMG consists of fine-grained spectrospatial information that can discriminate between different hand movement tasks. This finding is particularly interesting, because spectral features have not been considered successful in EMG signal classification in a traditional feature extraction setting [42]. A possible explanation for good results in our study is that the three-way classifiers can exploit detailed information from the entire spectrospatial structure of the sensors/components and can simultaneously avoid overfitting due to a low-rank assumption and regularization. In fact, our classification results suggest that *both* sparsity and low-rank constraints are needed to allow accurate classification and meaningful interpretation of EMG data: Clearly, relatively poor result of the classifier based on only the sparsity constraint (“Linear”) shows that sparsity alone is not sufficient for reliable classification. The low-rank constrained classifier (“TraceNorm”) yielded a realible performance, but it does not allow for simple interpretation of the relevant features because the solution is not sparse. Our sparse and bilinear classifier solves these limitations by combining both the constraints.

Related sparse and bilinear methods have been proposed by [43, 44]. However, our method is clearly different from such previous work. Our primary goal is not to reduce the rank of the matrix through sparsity (i.e., to remove the entire column of the parameter matrices) unlike in [43, 44], but to obtain sparse parameter vectors with only small number of nonzero elements. This is highly useful in the EMG analysis context, where our goal is to associate classification coefficients to the entire spatial patterns of the muscle activity. With too many non-zero coefficients, the interpretation of the final classifiers would be extremely difficult.

Recently, [45] suggested a bilinear modeling approach for hand movement classification from EMG signals. They first extracted features from four EMG electrodes placed around the forearm of the subjects, and then constructed a bilinear model to factorize the measured data into motion- and user-dependent factors. After this, they used the estimated motion-dependent variables and an ordinary SVM classifier to predict the movement category. Our approach is clearly different from this one, because we directly make class predictions on the basis of EMG data by using the bilinear model.

Previously, spatial filtering based on CSP was proposed prior to EMG signal classification [1]. Our new approach differs notably from that one in terms of neurophysiological insights it provides: The CSP-based approach tells which EMG sensors contain task-discriminative information, whereas the sparse bilinear approach provides a set of EMG activity patterns (characterized by their magnitude and phase) which is most discriminative in a given task. Hence, the two approaches can be used together in future to provide complementary information about the underlying physiological processes of muscles.

Many previous studies have used instantaneous ICA to model EMG signal activity. We estimated ICs in time-frequency domain to better capture characteristics of propagating MUAPs. Under real-world conditions, there are also non-stationaries present in the mixing which are caused e.g. by changing arm positions, movements of the sensor in relation to the muscles, or changing skin-conditions [46]. We did not explicitly model those non-stationarities, but the high classification accuracies regardless of a variation of the arm positions in our hand-movement experiment demonstrated that our methods are relatively robustness against this important source of non-stationarity.

It is also interesting to note that the 2D-LDA, which implements the “full” Fisher discriminant analysis using information in the covariance matrix, provided slightly lower overall accuracy than the simplified, or regularized, version given by our three-way DA. This is presumably because in the 2D-LDA, just as in the general formulation of Fisher LDA for two-way data, the covariance matrix needs to be inverted, or some equivalent tensor operation needs to be performed. Such inversion can be numerically highly unstable, and it can be subject to strong random fluctuations due to finite sample size.

Taken together, the proposed methods can efficiently utilize spectrospatial structure of the high-density EMG data to distinguish between six different hand movements with high accuracy. Furthermore, our investigations with finger lift data suggest that the methods provide classifiers that are interpretable and can thus give novel insights into EMG data. Therefore, our results hold high potential for prosthetics which use EMG to extract control information, as well as for medical diagnosis (cf. [46–48]). Moreover, our methods are not limited to EMG signal analysis, but they can be also applied to other types of biomedical data sets, such as functional brain imaging signals.
